# The role of the oncostatin M/OSM receptor β axis in activating dermal microvascular endothelial cells in systemic sclerosis

**DOI:** 10.1186/s13075-020-02266-0

**Published:** 2020-07-31

**Authors:** G. Marden, Q. Wan, J. Wilks, K. Nevin, M. Feeney, N. Wisniacki, M. Trojanowski, A. Bujor, L. Stawski, M. Trojanowska

**Affiliations:** 1 Arthritis Centre, Boston University School of Medicine, Boston University, 72 East Concord St, E-5, Boston, MA 02118 USA; 2 Department of Rheumatology and Endocrinology, Zhongnan Hospital of Wuhan University, Wuhan, China; 3 Immuno-Inflammation Therapeutic Area Unit, GlaxoSmithKline, Stevenage, UK

**Keywords:** OSM, OSMRβ, IL-6, Endothelial cells, SSc, FLI1, ERG

## Abstract

**Background:**

Scleroderma (SSc) is a rare autoimmune disease characterized by vascular impairment and progressive fibrosis of the skin and other organs. Oncostatin M, a member of the IL-6 family, is elevated in SSc serum and was recognized as a significant player in various stages of fibrosis. The goal of this study was to assess the contribution of the OSM/OSMRβ pathway to endothelial cell (EC) injury and activation in SSc.

**Methods:**

IHC and IF were used to assess the distribution of OSM and OSMRβ in SSc (*n* = 14) and healthy control (*n* = 7) skin biopsies. Cell culture experiments were performed in human dermal microvascular endothelial cells (HDMECs) and included mRNA and protein analysis, and cell migration and proliferation assays. Ex vivo skin organoid culture was used to evaluate the effect of OSM on perivascular fibrosis.

**Results:**

OSMRβ protein was elevated in dermal ECs and in fibroblasts of SSc patients. Treatments of HDMECs with OSM or IL-6+sIL-6R have demonstrated that both cytokines similarly stimulated proinflammatory genes and genes related to endothelial to mesenchymal transition (EndMT). OSM was more effective than IL-6+sIL-6R in inducing cell migration, while both treatments similarly induced cell proliferation. The effects of OSM were mediated via OSMRβ and STAT3, while the LIFR did not contribute to these responses. Both OSM and IL-6+sIL-6R induced profibrotic gene expression in HDMECs, as well as expansion of the perivascular PDGFRβ^+^ cells in the ex vivo human skin culture system. Additional studies in HDMECs showed that siRNA-mediated downregulation of FLI1 and its close homolog ERG resulted in increased expression of OSMRβ in HDMECs.

**Conclusions:**

This work provides new insights into the role of the OSM/OSMRβ axis in activation/injury of dermal ECs and supports the involvement of this pathway in SSc vascular disease.

## Introduction

Scleroderma also known as systemic sclerosis (SSc) is a complex chronic multisystem disease of unknown etiology that is characterized by early vascular damage with activation of the immune system, followed by skin and internal organ fibrosis [1]. The widespread structural changes of the microvasculature are well documented in SSc patients; however, many aspects of SSc vasculopathy, including the nature of the injury and the pathological consequences of injured endothelial cells (ECs), remain poorly understood. ECs play an important role in orchestrating tissue response to injury [2, 3]. In addition to the secretion of proinflammatory and profibrotic cytokines, ECs may also contribute to the perivascular extracellular matrix (ECM) remodeling by transitioning to mesenchymal cells through the process of endothelial to mesenchymal transition (EndMT), as well as through more direct interactions with fibroblasts [4]. Although, the presence of EndMT was reported in several animal models of inducible fibrosis, [5] as well as the skin and lungs of scleroderma patients [6], contribution of EndMT to the pathogenesis of fibrotic diseases remains controversial.

The IL-6 cytokine family encompasses a group of pleiotropic cytokines produced by a variety of cells in response to inflammatory stimuli [7]. This cytokine family shares a common signal transducer gp130 in the receptor complex. Members of the IL-6 family activate the JAK/STAT and MAPK signaling pathways and are involved in many biological processes including differentiation, hematopoiesis, cell proliferation, and cell survival [7]. Increased levels of IL-6 family members, including oncostatin M (OSM) and IL-6, have been reported in many pathological conditions characterized by chronic inflammation, vascular injury, and fibrosis including SSc [8]. Moreover, targeting IL-6 has been beneficial in many diseases including those characterized by ECM remodeling [9, 10]. OSM was shown to play an important role in various stages of the fibrotic process including inflammation and activation of fibroblasts [11, 12]. However, its role in activating ECs is still poorly explored, despite the fact that ECs express high levels of OSMRβ making them one of the primary targets of OSM. The goal of this study was to assess the effects of OSM on EC activation and the potential contribution of OSM signaling to SSc pathogenesis.

## Material and methods

### Human subjects

Upon informed consent and in compliance with the Institutional Review Board (IRB) for Human Studies, skin biopsies from the affected areas were obtained from nine patients with diffuse SSc, eight patients with limited SSc, and seven healthy donors. Patient characteristics are included from the affected areas were obtained from nine patients with diffuse SSc, eight patients with limited SSc, and seven healthy donors. Patient characteristics are included in Table 1.

**Table 1:** Healthy controls and Scleroderma patient’s data

Name	Sex	Age	Disease duration	Skin score	Endothelial cells/perivascular cells	Fibroblasts
HC1	F	42	–		+	–
HC2	F	28	–		++	+
HC3	F	52	–		+	–
HC4	M	28	–		+	–
Diffuse SSc 1	F	62	2 years	24	++	+
Diffuse SSc 2	F	47	1 year	15	+++	++
Diffuse SSc 3	F	47	8 years	15	+++	++
Diffuse SSc 4	F	72	1 year	34	++	++
Diffuse SSc 5	M	33	3 years	18	++	++
Diffuse SSc 6	M	36	7 years	35	+++	++
Diffuse SSc 7	M	61	5 years	54	+++	++
Limited SSc 1	F	42	2 years	3	+++	++
Limited SSc 2	F	52	2 years	3	++	+
Limited SSc 3	F	42	2 years	4	++	++
Limited SSc 4	F	68	1 year	0	+	+
Limited SSc 5	F	37	1 year	2	++	+
Limited SSc 6	F	52	5 years	16	++	++
Limited SSc 7	F	44	2 years	3	+++	+
**Biopsies used in IF**						
HC 4	F	63				
HC 5	M	44				
HC 6	F	50				
Diffuse SSc 8	F	60	5 years	12		
Diffuse SSc 9	M	62	2 months	23		
Limited SSc 10	F	74	4 years	0		

*F* female, *M* male

### Cells

Human dermal microvascular endothelial cells (HDMECs) were isolated from human foreskin as previously described [13, 14]. The human biological samples were sourced ethically, and their research use was in accord with the terms of the informed consent under an IRB/EC-approved protocol. Cells were cultured on bovine collagen-coated 6-well plates in EBM medium supplemented with 10% FBS, and EC growth supplement mix at 37 °C with 5% CO_2_ in air. All the experiments were performed on cells from early passages.

### siRNA transient transfections

HDMECs were transfected with siRNA specific to human OSMRβ, LIFR, ERG, and FLI1 (ON-TARGETplus SMART pool; GE Dharmacon, Lafayette, CO) or negative control siRNA at the concentration of 10 nM using Lipofectamine RNAiMAX Transfection Reagent (Thermo Fisher Scientific, Waltham, MA) according to the manufacturer’s protocol.

### Western blot

For Western blot, whole-cell extracts were prepared from HDMECs using lysis buffer with the following composition: 1% Triton X-100, 50 mmol/L Tris-HCl (pH 7.4), 150 mmol/L NaCl, 3 mmol/L MgCl_2_, 1 mmol/L CaCl_2_, proteinase inhibitor mixture (Roche), and 1 mmol/L phenylmethyl sulfonyl fluoride. Protein extracts were subjected to SDS-PAGE and transferred to nitrocellulose membranes. Membranes were incubated overnight with primary antibodies, washed, and incubated for 1 h with appropriate horseradish peroxidase-conjugated secondary antibody. After washing, visualization was performed by enhanced chemiluminescence (Pierce, Rockford, IL). Primary antibodies and concentrations are listed in Supplemental Table II.

### Quantitative RT-PCR analysis

Total RNA was isolated using TRIzol reagent (MRC, Inc., Cincinnati, OH). Real-time PCR assays were performed using the StepOnePlus Real-Time PCR system (Applied Biosystems, Foster City, CA). Briefly, 1 μg of total RNA was reverse transcribed with random hexamers using the Transcriptor First Strand complementary DNA Synthesis kit (Roche Applied Science, Indianapolis, IN) according to the manufacturer’s protocol. The amplification mixture (10 μl) contained 1 μl of complementary DNA, 0.5 μM of each primer, and 5 μl of SYBR Green PCR Master Mix. The primers are listed in Supplementary Table I. Relative changes in the levels of genes of interest were determined by the 2^−ΔΔCT^ method.

### Immunofluorescence staining on adherent cell cultures

For immunofluorescence, cultured HDMECs were grown on collagen-coated cover slips. Cells were treated with OSM and IL-6+sIL6R for 48 h and 72 h, or siRNA for ERG and FLI1. Cells were fixed with 4% paraformaldehyde for 15 min. Non-specific protein binding was blocked with 3% BSA for 1 h. Next, cells were incubated at 4 °C overnight with primary antibody. After washing, cell cultures were incubated with appropriate fluorophore-conjugated secondary antibody (Invitrogen, Carlsbad, CA) for 1.5 h. Skin biopsies were embedded in OCT and fixed in acetone:methanol (1:1). Sections were blocked in 3% BSA for 1 h, before the addition of primary antibodies diluted in 1% BSA. After washing, sections were incubated in appropriate fluorophore-conjugated secondary antibodies for 45 min. Cells and biopsy sections were mounted on slides using Vectashield with DAPI (Vector Laboratories, Burlingame, CA) and examined using a FluoView FV10i confocal microscope system (Olympus, Center Valley, PA) at 488 nm (green), 594 nm (red), and 405 nm (blue). Secondary Alexafluor antibodies (Invitrogen, Carlsbad, CA) were used for each stain. Primary antibodies and concentrations are listed in Supplemental Table II.

### Migration and proliferation assay

Migration and proliferation were examined using the Essen BioScience IncuCyteTM Live-Cell Imaging system. Briefly, HDMECs were plated on an ImageLock 96-well plate and grown to 100% confluence (for the migration) or 5–10% confluence (for the proliferation) cells were treated additionally with 10, 50, and 100 ng/ml of OSM or IL-6 and sIL-6R images were captured every 3 h for a total of 50 h. Area under curves was measured using the GraphPad Prism software.

### Immunohistochemistry

Immunohistochemistry was performed on formalin-fixed, paraffin-embedded skin tissue sections. Briefly, sections (5-μm thick) were deparaffinized with Histo-Clear (National Diagnostics, Atlanta, GA), and rehydrated through a graded series of ethanol. For OSMRβ, endogenous peroxidase was blocked by incubation in 3% hydrogen peroxide for 30 min, followed by normal blocking serum for 1 h. The sections were then incubated overnight at 4 °C with primary antibody diluted in blocking buffer, followed by incubation for 30 min with appropriate polymer detection kit. Immunoreactivity was visualized with NovaRED (Vector Laboratories, Burlingame, CA). For OSM, antigen retrieval was performed using 1 mM Tris-EDTA pH 9.0. Sections were blocked with TBS containing 5% normal horse serum and then incubated overnight at 4 °C with primary antibody. Appropriate polymer detection kit was used for a subsequent 30-min incubation. Immunoreactivity was visualized with diaminobenzidine (Vector Laboratories, Burlingame, CA), and sections were counterstained with hematoxylin. For double staining, slides were prepared as described and incubated with primary antibody overnight. Subsequent appropriate polymer detection kit was used, and immunoreactivity was visualized with NovaRED (Vector Laboratories, Burlingame, CA). Quenching was achieved with 3% hydrogen peroxide. Sections were incubated in a primary antibody. Appropriate polymer detection kit was used, and immunoreactivity was visualized with high depth blue (Enzo Life Sciences, Farmingdale, NY). Images were collected using a microscope (BH-2; Olympus, Center Valley, PA). ImmPRESS HRP Polymer Detection Kits (Vector Laboratories, Burlingame, CA) were used for each stain. Primary antibodies and concentrations are listed in Supplemental Table II.

### Histologic assessment

The OSMRβ staining intensity for immunohistochemistry was scored semiquantitatively. The staining intensity (1, negative or weak staining; 2, moderate staining; and 3, strong staining) was evaluated in six randomly selected fields in subcutaneous area. Then a semiquantitative score per sample was generated by calculating the average of the six intensity scores per sample. Semiquantitative analysis was performed by two independent blinded researchers.

### Gomori’s trichrome staining

Gomori’s trichrome staining was used to detect collagen deposition. The skin samples were fixed in 4% paraformaldehyde for 24 h and then processed for paraffin embedding. Staining was performed on 5-μm-thick paraffin sections following the manufacturer’s instructions (Chromaview, Dublin, OH, Gomori’s Trichrome Blue Collagen Kit cat# S7440-19). Collagen fibers were stained blue, nuclei were stained black, and the background was stained red.

### Human skin organoid culture ex vivo

We utilized the previously described dermal ex vivo organoid culture technique [15]. The human biological samples were sourced ethically, and their research use was in accord with the terms of informed consent under an IRB/EC-approved protocol. Briefly, dermal biopsy punches (6 mm) obtained from foreskins were placed onto nitrocellulose membranes, to avoid contact with plastic or matrigel, and treated with human recombinant OSM (obtained from GlaxoSmithKline, Stevenage, UK) and IL-6+sIL-6R for 14 days. The medium was changed, and the OSM and IL-6+sIL-6R treatments were repeated every 48 h. At day 14, tissue biopsies were collected for IHC analysis.

### Statistical analyses

Data were analyzed by Student’s *t* test or Mann-Whitney *U* test where appropriate. The level for statistical significance was set at *p* ≤ 0.05.

## Results

### OSMRβ is elevated in the endothelial cells and fibroblasts of limited and diffuse SSc skin biopsies

**Fig. 1:**
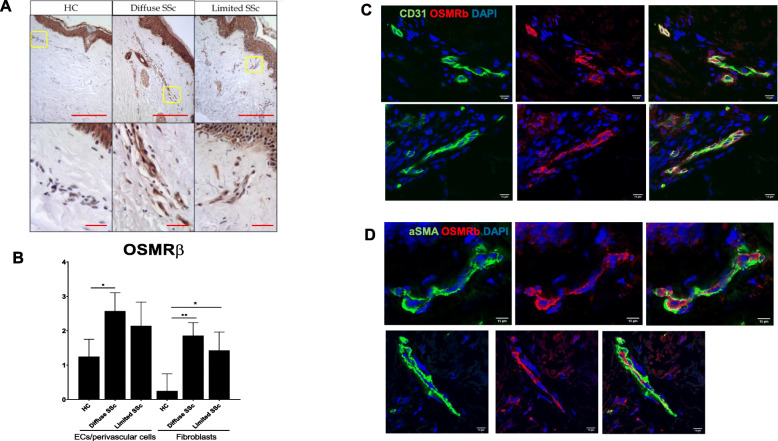
Distribution of OSMRβ in human skin biopsies from healthy controls and SSc patients. **a** IHC staining of OSMRβ was performed on paraffin sections from the skin of healthy controls and SSc patients. **b** The bar graph represents the staining intensity in endothelial/perivascular cells and fibroblasts (0, no staining; 1, negative or weak staining; 2, moderate staining; and 3, strong staining). Two hundred micrometers for original magnification × 4 and 25 μm for original magnification × 40 images. Results are shown as mean ± SD (Mann-Whitney *U* test, *p < 0.05, **< 0.01). **c** Double IF staining of CD31 and OSMRβ in two representative SSc skin biopsies (*n* = 3 SSc and 3 HC samples). **d** Double IF staining of αSMA and OSMRβ in two representative SSc skin biopsies. Scale bar 15 μm for original magnification × 60

OSMRβ was recently identified as a prognostic biomarker that correlates with progression of the skin disease in patients with diffuse systemic sclerosis (dcSSc) [16]. To illustrate the distribution of OSMRβ and OSM in SSc skin, we performed immunohistochemical (IHC) staining on biopsies from diffuse and limited patients. As shown in Fig. 1a, we observed increased expression of OSMRβ in the skin vessels of SSc patients as compared to healthy control skin. Semiquantitative scoring of the staining intensity demonstrated increased OSMRβ levels mostly in endothelial/perivascular cells and fibroblasts of SSc patients (Fig. 1b). In contrast, OSM protein, which was also detected in endothelial cells and fibroblasts, was comparable in SSc and HC skin biopsies (Supplemental Figure 1). Double immunofluorescence of OSMRβ and CD31 confirmed the presence of OSMRβ on ECs (Fig. 1c). OSMRβ did not appear to co-localize with αSMA in the skin (Fig. 1d). These results suggest that increased expression of OSMRβ on ECs could contribute to the process of vasculopathy in the skin of SSc patients.

### OSM regulates mRNA levels of proinflammatory genes in HDMECs

**Fig. 2:**
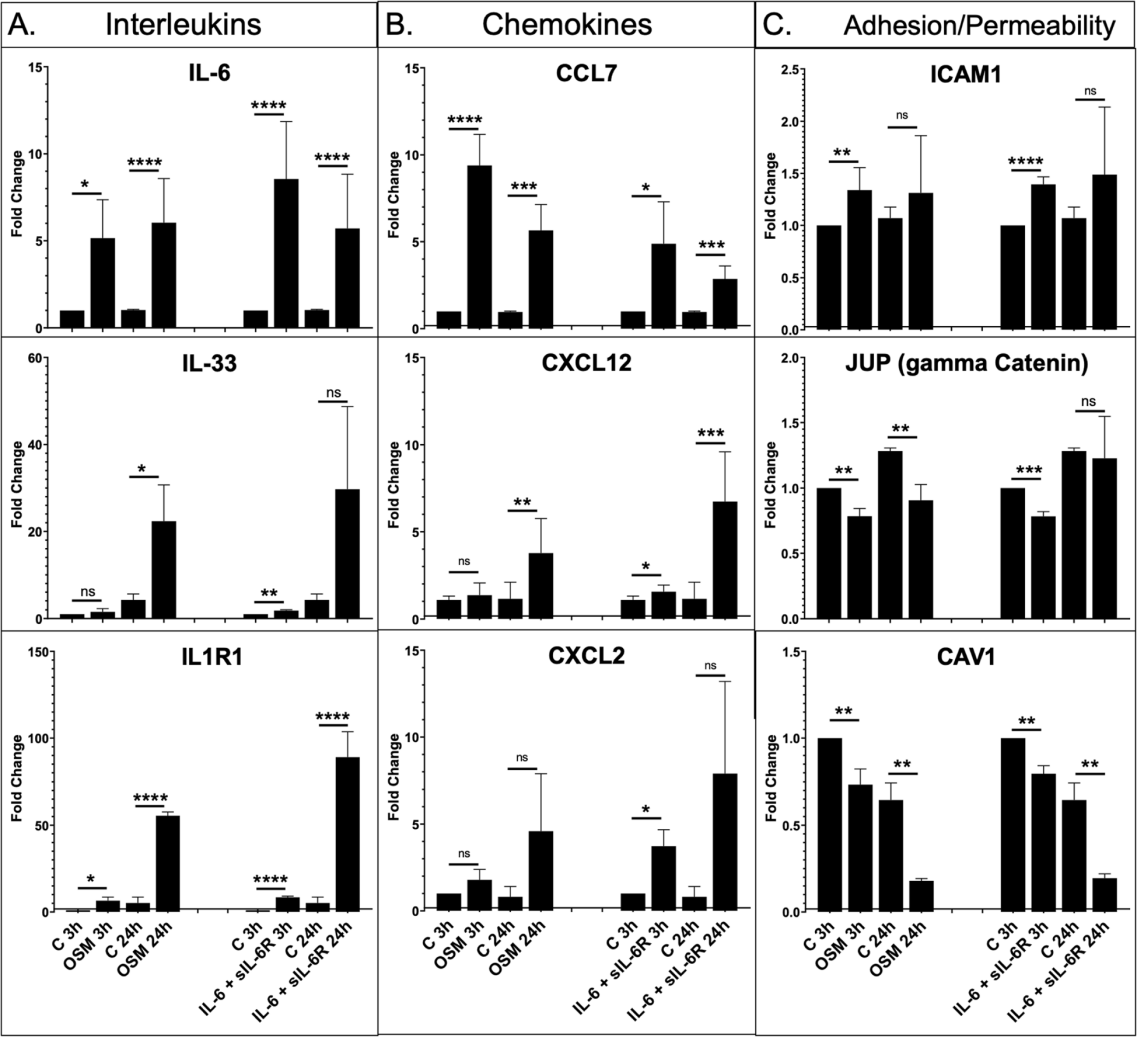
Effect of OSM and IL-6+sIL-6R on the mRNA levels of inflammatory genes in HDMECs. mRNA levels of interleukins (**a**), chemokines (**b**), and adhesion/permeability genes (**c**) were analyzed by quantitative PCR, *n* = 3. Student’s *t* test *p < 0.05, **p < 0.01, ***p < 0.001 ****p < 0.0001

OSM was previously shown to regulate expression of proinflammatory cytokines and adhesion molecules in ECs [17, 18]. To assess the effect of OSM on the inflammatory phenotype of HDMECs, we examined the gene expression of selected interleukin, chemokine, and adhesion molecule genes by real-time PCR. Cells were treated with human recombinant OSM (10 ng/ml) for 3 and 24 h. Human recombinant IL-6 (100 ng/ml) was used for comparisons. Since HDMECs have very low expression of IL-6R, addition of soluble IL-6R (sIL-6R) was required to initiate IL-6 signaling. A rapid increase in IL-6 mRNA levels in cells treated with OSM and IL-6+sIL-6R occurred at 3 h and remained high at 24 h (Fig. 2a). Expression levels of other IL-6 family members, including LIF and OSM were unchanged (data not shown). We also observed increased mRNA levels of IL33 and its receptor IL1R1 in cells treated with OSM and IL-6+sIL-6R for 3 h and 24 h (Fig. 2a). Among the chemokines, increased mRNA levels of CCL7 (also known as MCP3), CXCL12, and CXCL2 were observed in response to both treatments at 3 h and 24 h (Fig. 2b). The expression of adhesion molecule ICAM-1 was increased only at the 3 h timepoint in cells treated with OSM and IL-6+sIL-6R (Fig. 2c). OSM seemed to be a more potent inducer of CCL7 than IL-6+sIL-6R (Fig. 2b). Furthermore, induction of IL33 was sustained at the 24 h timepoint by OSM, while high variability with IL-6+sIL-6R-treated HDMECs resulted in an increase that was not statistically significant (Fig. 2a). Expression of other adhesion molecules, including ICAM-2 and VCAM-1 were unchanged (data not shown). Interestingly, JUP (also known as plakoglobin or gamma catenin) and CAV1 mRNA levels were gradually decreasing over time with both treatments (Fig. 2c). These data suggest that both OSM and IL-6+sIL-6R can induce a proinflammatory phenotype in HDMECs; however, IL-6 required a 10× higher concentration and the addition of the sIL-6R to achieve comparable results.

### OSM stimulates transition to a mesenchymal phenotype in HDMECs

**Fig. 3:**
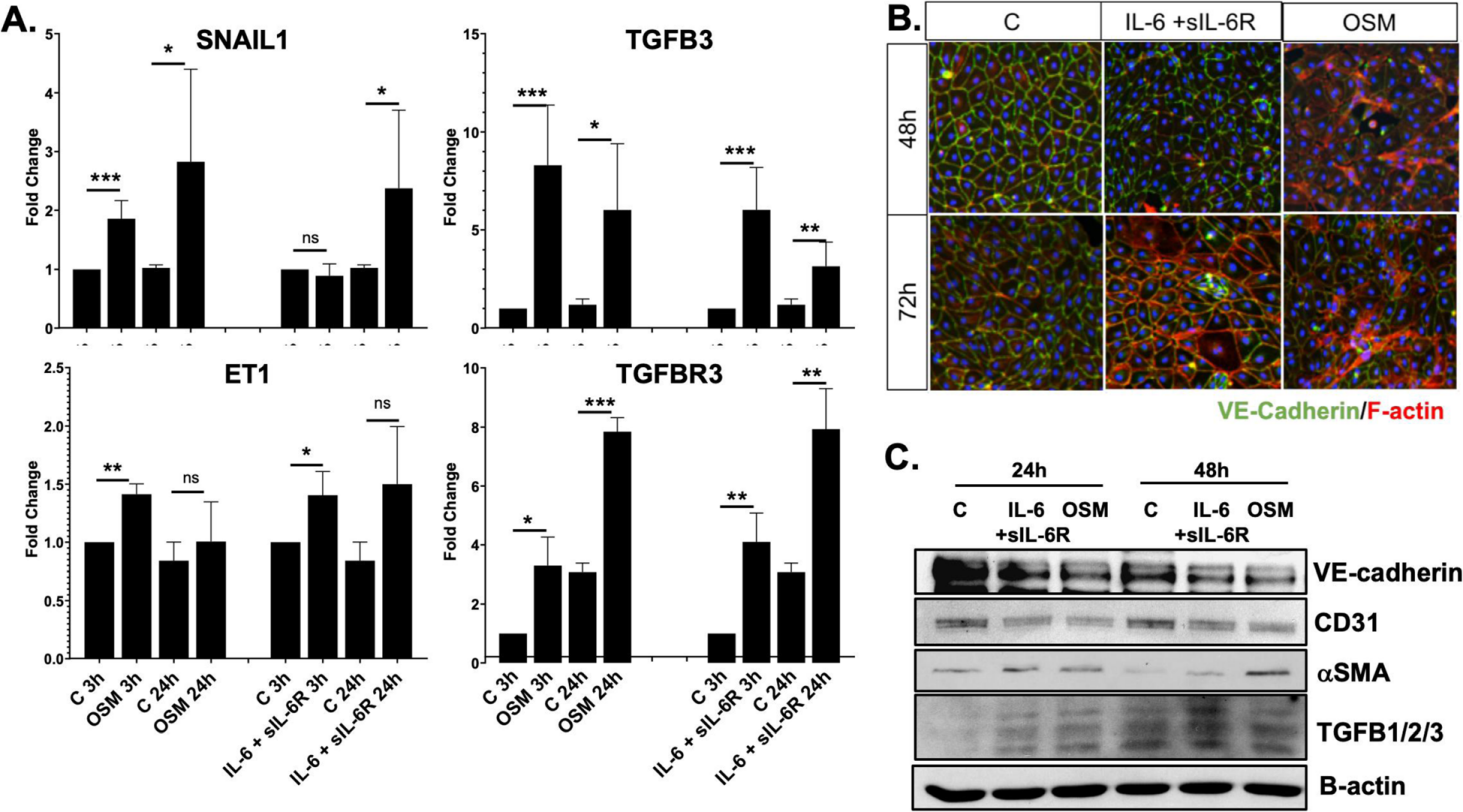
OSM and IL-6+sIL-6R stimulate transition to mesenchymal phenotype in HDMECs. **a** mRNA levels of EndMT genes were analyzed by quantitative PCR, *n* = 3. Student’s *t* test *p < 0.05, **p < 001, ***p < 0.0001. **b** Immunofluorescence staining of VE-cadherin (green) and phalloidin (red) in the HDMECs culture treated with OSM and IL-6+sIL-6R. Representative images are shown from 3 cell lines. **c** Representative western blot of VE-cadherin, CD31, αSMA, and TGFβ123 in HDMECs treated with OSM and IL-6+IL-6R for 24 and 48 h. *N* = 2

Previous studies have shown that bovine aortic endothelial cells (BAEC) treated with OSM became spindle-shaped and exhibited increased proliferation and migration [19]. Likewise, we found that HDMECs treated with human recombinant OSM (10 ng/ml) showed a statistically significant increase in mRNA levels of selected EndMT genes, including SNAIL1, TGFβ3, ET-1, and TGFβ3R at 3 h and 24 h when compared to the controls (Fig. 3a). Treatment with human recombinant IL-6+sIL-6R had similar effects on the mRNA levels of TGFβ3, ET-1, and TGFβ3R; however, significant changes to mRNA levels of SNAIL1 were only observed at 24 h (Fig. 3a).

To determine the effect of OSM and IL-6+sIL-6R on EC morphology, we performed double-fluorescence staining for VE-cadherin and phalloidin. HDMECs treated with OSM showed decreased VE-cadherin staining as well as elongated F-actin stress fibers at 48 h and 72 h (Fig. 3b). In contrast, treatment with IL-6+sIL-6R showed similar changes only at 72 h (Fig. 3b). Western blot analysis confirmed decreased levels of endothelial markers such as VE-cadherin and CD31 and increased levels of αSMA and TGFβ1, -2, -3 at 24 h and 48 h timepoints in cells treated with OSM or IL-6+sIL-6R (Fig. 3c). Together, these data suggest that both OSM and IL-6+sIL-6R can induce morphological EndMT-like changes in HDMECs with OSM acting more rapidly when compared to IL-6+sIL6R.

Because cells undergoing EndMT could acquire a more migratory phenotype, we assessed the effect of OSM and IL-6+sIL-6R on HDMEC migration using the scratch assay provided by the Essen BioScience IncuCyteTM Live-Cell Imaging system. HDMECs were treated with OSM and IL6+sIL-6R at the concentrations of 10, 50, and 100 ng/ml for 50 h. Cells stimulated with OSM showed significantly increased migration at the lowest dose, while the higher doses had no additional effect. In contrast, in cells treated with IL-6+sIL-6R, we only observed a trend toward increased migration, which was not statistically significant (Supplementary Figure 2). It may be relevant to the pro-migratory effects of OSM that plasminogen activation system-related genes, urokinase plasminogen activator (PLAUR), and tissue plasminogen activator (PLAT), were induced by OSM only [20] (Suplementary Figure 2).

**Fig. 4:**
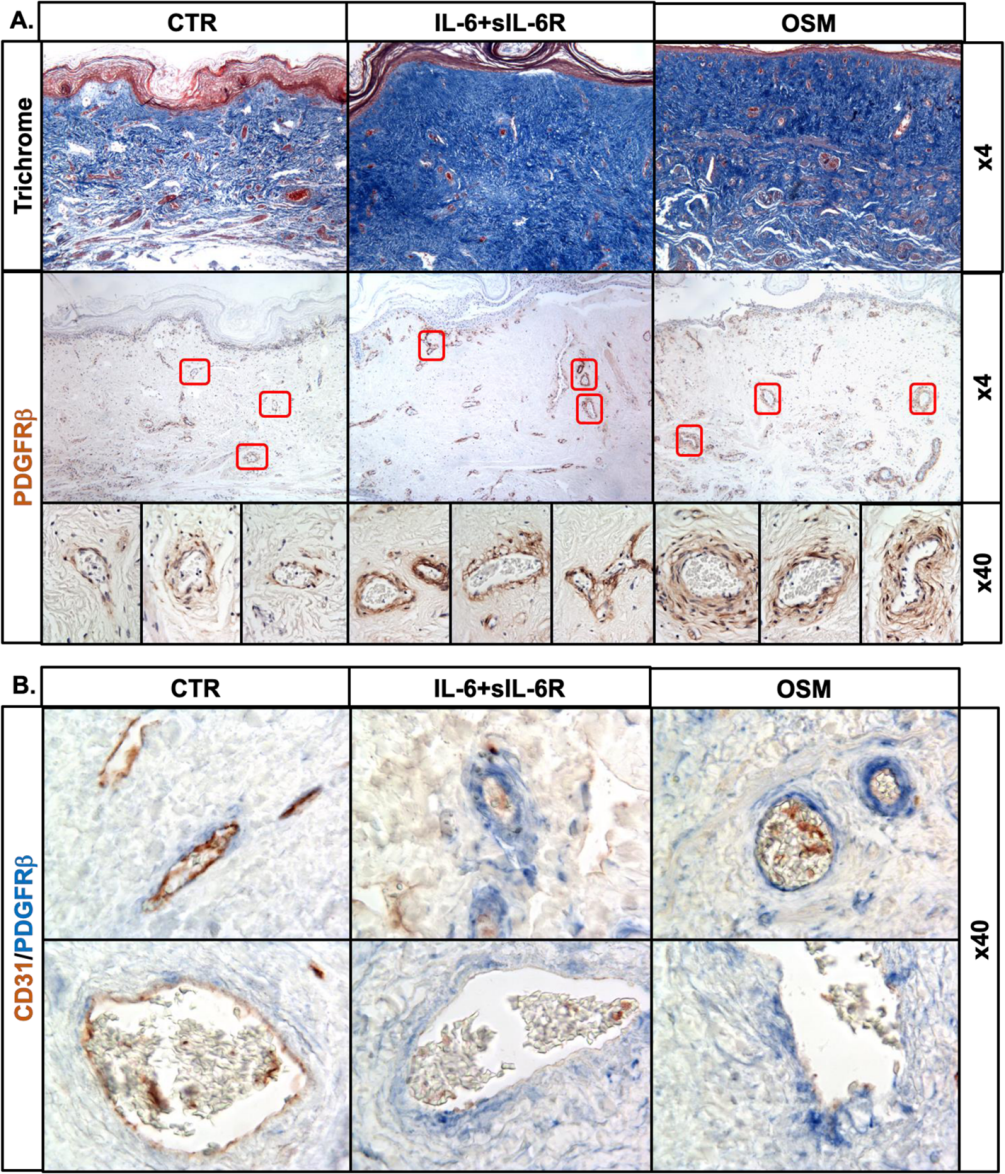
Effect of OSM and IL-6+sIL-6R on human skin organoid cultures. Dermal biopsy punches (6 mm) obtained from foreskins were placed onto nitrocellulose membranes and treated with OSM or IL-6+IL-6R for 14 days. Stainings were performed on paraffin sections. **a** Trichrome staining and IHC staining of PDGFRβ. **b** Double IHC of PDGFRβ/CD31. Two hundred micrometers for original magnification × 4 and 25 μm for original magnification × 40 images, *n* = 3. **c** Effect of OSM and IL-6+sIL-6R on the mRNA levels of profibrotic genes in HDMECs. mRNA levels of profibrotic genes were analyzed by quantitative PCR, *n* = 3. *p < 0.05, **p < 0.01, ***p < 0.001

We next evaluated the effect of OSM and IL-6+sIL-6R on HDMECs proliferation. Cells were treated with OSM and IL-6+sIL-6R at the concentrations of 10, 50, and 100 ng/ml for 50 h. Both OSM and IL-6+sIL-6R significantly induced proliferation of HDMECs; however, IL-6+IL-6R increased cell proliferation at lower concentrations than OSM (Supplementary Figure 2B). OSM and IL-6+sIL-6R exhibited similar behavior in a capillary tube formation assay in the presence of 2.5% FCS; however, neither cytokine was able to efficiently induce tube formation in 1% FCS (Supplementary Figure 2C). These data suggest that OSM compared to IL-6+sIL-6R is a more potent inducer of HDMECs migration. In contrast, IL-6+sIL-6R, although a weak stimulator of cell migration, potently induced proliferation of HDMECs. Together these data demonstrated an important role of OSM in modulating the function of HDMECs.

### OSM induces a profibrotic response in human skin organoid cultures

To further investigate the effects of OSM and IL-6+sIL-6Ra on vascular injury, we employed an ex vivo human skin culture system, which more closely mimics the in vivo environment. OSM or IL-6+sIL-6R-treated tissue explants showed increased collagen deposition as well as an increased number of PDGFRβ^+^ cells around the vessels (Fig. 4a). To characterize those vessels, we performed double immunostaining for PDGFRβ and CD31. As shown on Fig. 4b, in tissues treated with OSM or IL-6+sIL-6R, vessels with an increased number of PDGFRβ positive cells also showed decreased expression of CD31. Moreover, we observed increased expression of phosphorylated STAT3 in the vessels and in numerous stromal cells in OSM and IL-6+sIL-6R-treated skin as compared to controls (Supplementary Figure 3A). Interestingly, activation of PDGFRβ was only observed around the blood vessels, but not lymphatic vessels as illustrated by the double staining for PDGFRβ/podoplanin (PDPN) (Supplemental Figure 3B). These observations are consistent with the expansion of the perivascular mesenchymal stromal cells during fibrosis [21].

To gain additional insights into the profibrotic effects of OSM, we assessed the expression of additional profibrotic genes. Cells treated with OSM or IL-6+sIL-6R, showed decreased expression of FGFR1 and increased expression of FAP, POSTN, and TIMP1 (Supplementary Figure 2). Also, CHI3L1 (YKL-40), a protein associated with fibrosis that has been implicated in SSc lung and skin fibrosis, was highly elevated by OSM, and to a lesser degree by IL-6+sIL-6R [22–25] (Supplementary Figure 2). Notably, increased expression of hyaluronan synthase (HAS2) and decreased expression of Wnt pathway inhibitor Dkk1 were only observed in cells stimulated with OSM (Supplementary Figure 2). HAS2 has been shown to regulate EndMT during cardiac valve formation [26]. Furthermore, elevated expression of HAS2 by lung fibroblasts promoted severe lung fibrosis [27]. Activation of the Wnt pathway has also been implicated in the process of EndMT [28], and downregulation of Dkk1 has been shown in SSc skin in vivo and in cultured SSc fibroblasts [29, 30].

### OSM-induced EC activation is mediated primarily by OSMRβ and depends on STAT3 phosphorylation

**Fig. 5:**
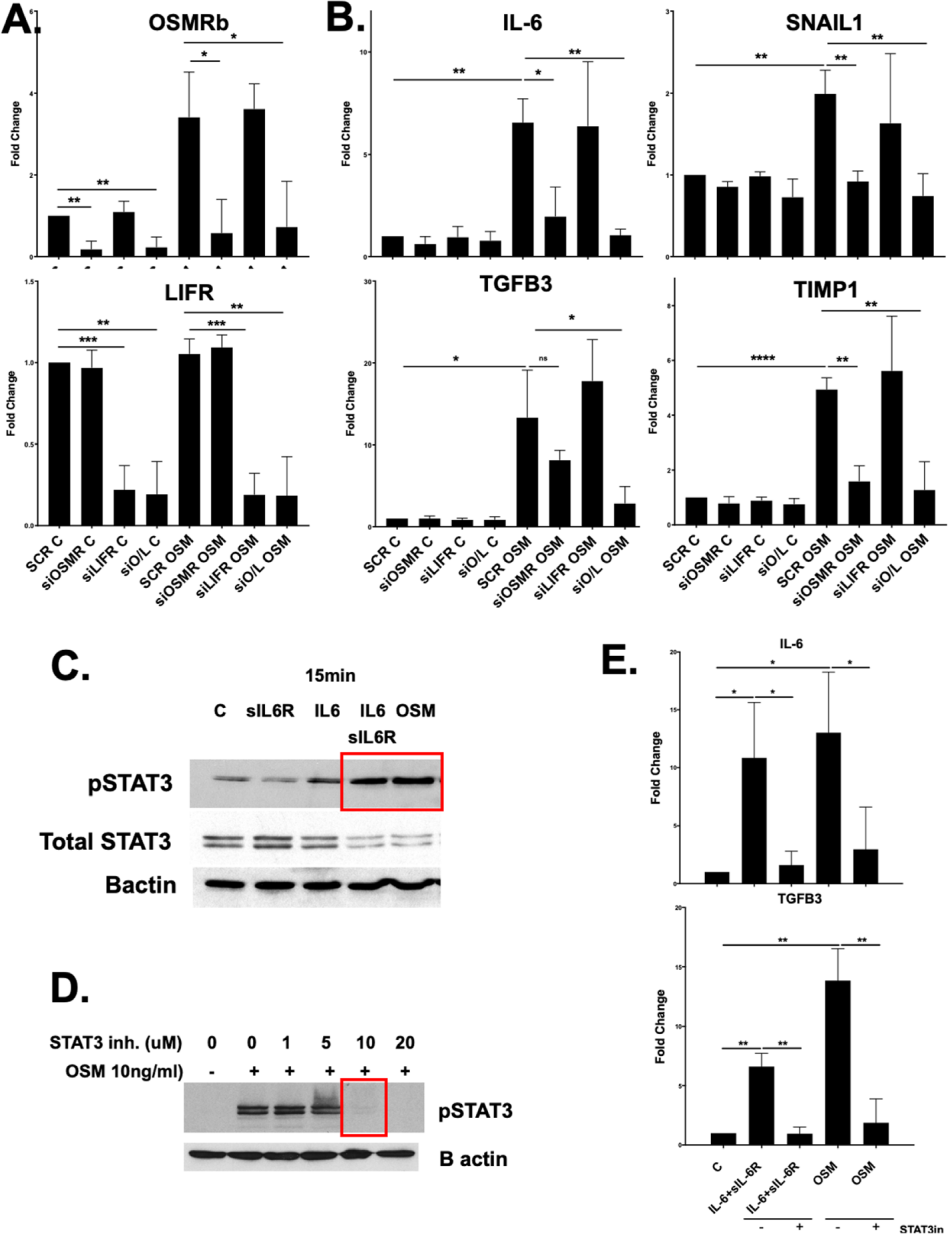
OSMRβ mediates OSM-induced EC activation in HDMECs. **a** Effect of OSMRβ and LIFR inhibition on OSM-induced mRNA levels. HDMECs were transfected with specific siRNA against OSMRβ, LIFR separately, or together for 24 h and then treated with OSM for another 24 h. **b** mRNA levels of TGFβ3, SNAIL1, IL-6, and TIMP1 were analyzed by quantitative PCR, *n* = 3. *p < 0.05, **p < 0.01, ***p < 0.001, ****p < 0.0001. **c-e** Effect of STAT3 inhibition on OSM-induced mRNA levels. HDMECs were pretreated with an inhibitor of STAT3 (BP-1-102) and then treated with OSM for 3 h and 24 h. mRNA levels of IL-6, TGFβ3, and TIMP1 were analyzed by quantitative PCR, *n* = 3. *p < 0.05, **p < 0.01

In humans, OSM signaling is initiated by binding of OSM to its specific type I receptor complex (LIFRβ/gp130**)** or type II receptor complex (OSMRβ/gp130**).** To determine which receptor is responsible for the OSM-induced phenotype in HDMECs, cells were treated with SCR, OSMRβ siRNA, LIFR siRNA, or both for 48 h and then stimulated with OSM for another 3 h. Cells treated with siOSMRβ, siLIFR, and both showed around 80% efficiency in downregulating these genes (Fig. 5a). Treatment with OSMRβ siRNA significantly decreased expression of IL-6, SNAIL1, and TIMP1, but only partially blocked the expression of OSM-induced TGFβ3 (Fig. 5b). In contrast, treatment with LIFR siRNA had no significant effect on the OSM-induced mRNA levels of any of these genes (Fig. 5b). Treatment with both OSMRβ/LIFR siRNA completely blocked the OSM-induced mRNA levels of all tested genes (Fig. 5b). This data suggests that OSM induces activation of HDMECs primarily via OSMRβ.

STAT3 is a transcription factor that is activated by IL-6 family cytokines, including OSM. The levels of the activated (phosphorylated) form of STAT3 are elevated in the skin and lung of SSc patients, suggesting that it is involved in SSc pathogenesis [31, 32]. HDMECs treated with OSM or IL-6+sIL-6R showed increased phosphorylation of STAT3 (Fig. 5c). A specific inhibitor of STAT3, BP-1-102 (10uM), completely blocked the STAT3 phosphorylation (Fig. 5d). To determine if OSM and IL-6+sIL-6R-induced phenotype is STAT3 dependent, we pretreated HDMECs with BP-1-102 for 1 h, and then treated with OSM and IL-6+sIL-6R for another 3 h and 24 h. BP-1-102 pretreatment reversed the OSM- and IL-6+sIL-6R-induced mRNA levels of IL-6 and TGFβ3 (Fig. 5e). In contrast, inhibitors of TGFβ (SB431542), ERK (SCH772984), and WNT (ICG-001) signaling pathways had no effect on the OSM- and IL-6+sIL-6R-induced gene expression (data not shown). These results suggest that OSM and IL-6+sIL-6R can activate ECs directly via STAT3 phosphorylation, independent of TGFβ, WNT, and ERK signaling.

### OSMRβ expression in HDMECs is regulated by FLI1 and ERG

**Fig. 6:**
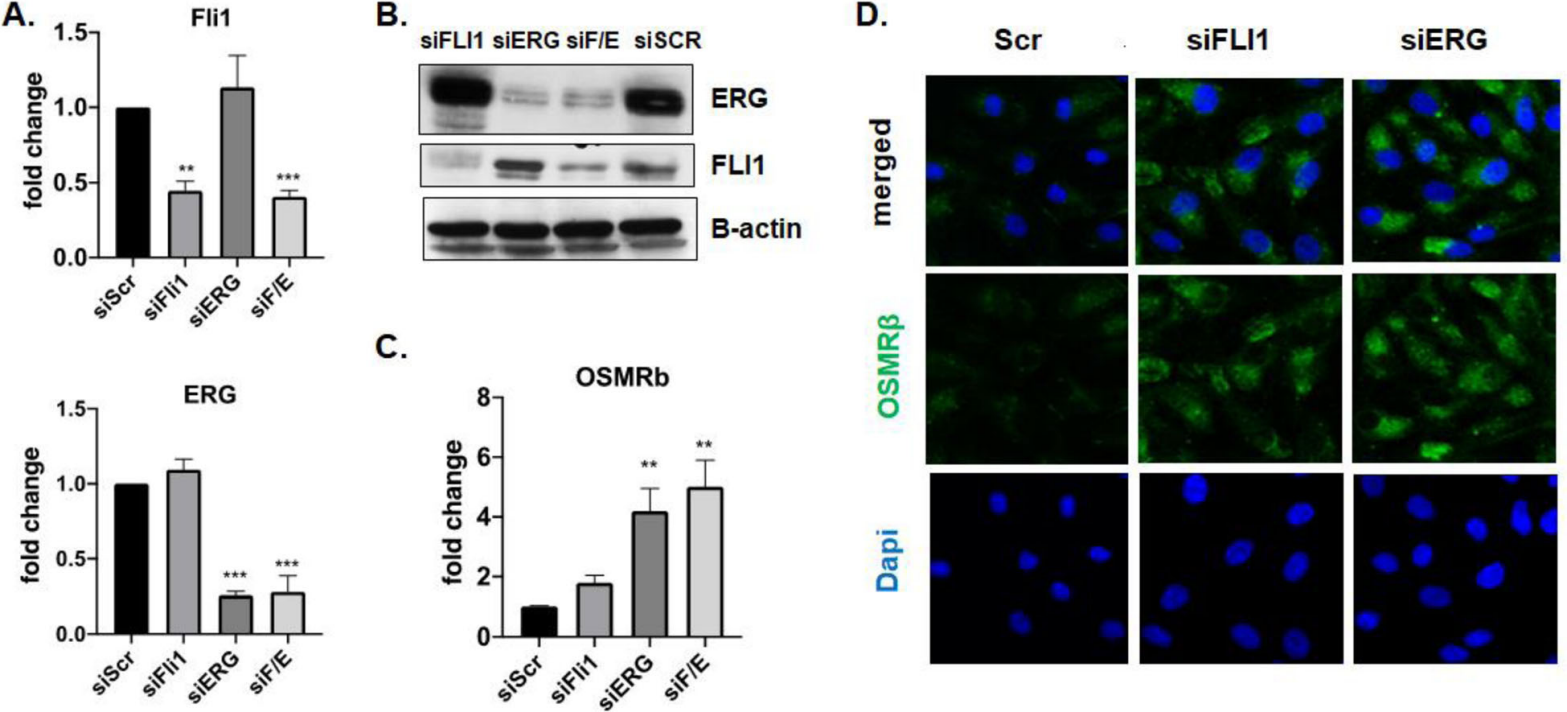
FLI1 and ERG regulate OSMRβ expression in HDMECs. mRNA levels of FLI1, ERG (**a**), and OSMRβ (**c**) were analyzed by quantitative PCR, *n* = 3. *p < 0.05, **p < 0.01, ***p < 0.001. **b** Representative western blot of FLI1 and ERG in HDMECs treated with siFLI1, siERG, and siSCR oligos for 48 h. *N* = 3. **d** Immunofluorescence staining of OSMRβ (green) in the HDMECs culture treated with OSM and IL-6+IL-6R. Representative images are shown from 3 cell lines

In the course of this study, we noticed that many of the effects of OSM/IL-6 on HDMECs, including downregulation of VE-cadherin and CD31, and upregulation of the profibrotic and proinflammatory genes were similar to those previously attributed to the deficiency of FLI1 [33–35], thus raising the possibility that FLI1 may mediate some of the functional effects of OSM/IL-6. However, OSM/IL-6 did not affect FLI1 protein levels, suggesting that those cytokines act independently of FLI1. Since FLI1 and its close homolog, ERG, are known to suppress inflammatory responses in ECs and the expression of both factors have been shown to be reduced in SSc ECs [33, 34], we next asked whether FLI1 or ERG could be involved in regulating the expression of OSMRβ. Depletion of either FLI1 or ERG led to increased mRNA and protein levels of OSMRβ, suggesting that the lower protein levels of these transcription factors in SSc vasculature may, at least in part, contribute to the increased expression of OSMRβ in SSc dermal ECs (Fig. 6).

## Discussion

There is increasing evidence linking IL-6 to endothelial dysfunction and vascular hypertrophy, as well as fibrosis, including SSc [36, 37]; however, the contribution of other IL-6 family members to these pathological processes, especially the activation of ECs, remains relatively understudied. Because OSM has also been implicated in SSc pathogenesis [38, 39], this study investigated how OSM influences HDMECs. We showed that the effects of OSM were comparable to that of IL-6/IL-6Rα, with both cytokines inducing a proinflammatory and profibrotic phenotype in HDMECs and in an ex vivo skin culture system. We further demonstrated that blocking the OSMRβ or STAT3 phosphorylation reversed the OSM-induced phenotype.

STAT3, a transcriptional effector of the JAK/STAT signaling pathway, regulates many cellular processes including proliferation, migration, apoptosis, and differentiation [40]. STAT3 can be activated by proinflammatory cytokines including members of IL-6 family, IL-6, and OSM [41, 42]. Persistent activation of STAT3 was observed in many diseases characterized by chronic inflammation and fibrosis including SSc [26]. In endothelial cells STAT3 activation was mostly linked to increased expression of adhesion molecules including E-selectin, P-selectin, and VCAM [43, 44]. In our experiments, OSM-induced ICAM1 and junction plakoglobin JUP; however, it did not affect expression of E- and P-selectin or VCAM.

Activation of OSM signaling is strongly related to the expression levels of its receptors. It was previously shown that in fibroblasts and epithelial cells OSM can regulate the synthesis and turnover of OSMRβ and LIFRβ by ligand-induced receptor degradation as well as by a compensatory mechanism of enhanced regulation of their mRNA levels [45]. In HDMECs, OSM can induce mRNA levels of OSMRβ, but not LIFRβ (data not shown), suggesting the initiation of the compensatory mechanism. Moreover, our data indicate that the OSM-induced phenotype is primarily mediated by OSMRβ in HDMECs.

Endothelial cells play a crucial role in inflammatory processes by maintaining the vessel integrity and immune cell trafficking. Excessive endothelial cell activation in chronic inflammatory settings can lead to EC dysfunction and development of a broad spectrum of human diseases [46, 47]. Here, we show that SSc dermal ECs expressed high levels of OSMRβ together with its cognate ligand, OSM, suggesting that the autocrine OSM/OSMRβ signaling could contribute to vascular inflammation in SSc. We further showed that depletion of transcription factors ERG and FLI1 in HDMECs led to the increased expression of OSMRβ, consistent with the role of these factors in suppressing vascular inflammation. Although, OSM did not affect FLI1 or ERG expression in HDMECs, previous studies have shown that various inflammatory mediators, including IFN-α, TLR ligands, and CXCL4, as well as profibrotic ligands TGFβ and ET-1, decreased protein levels of FLI1 in ECs [33, 48–50]. Similarly, ERG expression was downregulated in ECs in response to proinflammatory factors, including TNF-α, IL-1β, and LPS [51].

Both IL-6 and OSM were previously implicated in tissue fibrosis either by activating other profibrotic cytokines, or directly, by regulating fibroblast activation and ECM turnover. IL-6KO mice were protected from bleomycin-induced lung fibrosis [52]. Moreover, blockade of IL-6R resulted in decreased fibroblasts activation and alleviated bleomycin-induced skin fibrosis [53]. Similar observations were made for OSM. In vivo, OSM displayed profibrotic properties in different organs including the lung [12, 54], heart [55], and liver [56]. Blocking OSM was shown to ameliorate fibrosis in these organs. Similarly, blocking STAT3 with a specific inhibitor ameliorated fibrotic responses in the animal models of lung and skin fibrosis [31]. Notably, profibrotic effects of OSM in vivo are independent of TGF β and IL-4/IL-13 signaling pathways [12, 57]. However, a recent study using BALB/c mice has implicated IL-13-dependent accumulation of fibrocytes during OSM-induced lung fibrosis [54]. Consistent with these findings, we observed increased collagen deposition in ex vivo skin cultures. Notably, administration of OSM resulted in expansion of perivascular PDGFRβ^+^ cells. Increased presence of PDGFRβ^+^ cells was previously observed in the perivascular regions in the skin of early SSc patients and was suggested as a source of myofibroblasts [58]. In our ex vivo model, expansion of the PDGFRβ^+^ cells could be caused either by OSM directly stimulating proliferation of these cells or by injured endothelial cells activating these perivascular mesenchymal cells in a paracrine manner. It would be necessary to perform more experiments to answer this question.

A phase 2 clinical trial of IL-6Rα blocking antibody (Tociluzimab, TCZ) in SSc patients was recently completed and has shown only a trend of benefit for primary end point, mRSS [59]. However, dermal fibroblasts explanted from the TCZ-treated patients have shown complete reversal of their activated phenotype [60], the basis of these contradictory results is currently not clear but may suggest that IL-6Rα blockade affects only a subset of fibroblasts present in the skin in vivo. The explanted fibroblasts may not fully capture the heterogeneous population of collagen-producing cells in the fibrotic lesions. It remains an open question whether blockade of OSM would be more efficacious. A clinical trial targeting OSM in patients with SSc is currently ongoing (https://clinicaltrials.gov/ct2/show/NCT03041025).

## Conclusions

In summary, OSM signaling may play an important role during vessel degeneration and fibrosis in scleroderma. Blocking the OSM/OSMRβ pathway or inhibiting the STAT3 pathway could serve as a potential therapy for patients with scleroderma.

## Supplementary information

**Additional file 1: Supplementary Figure 1.** Distribution of OSM in human skin biopsies from healthy controls and SSc patients. **A.** IHC staining of OSM was performed on paraffin sections from the skin of three SSc patients and three healthy controls 50 μm scale bar for original magnification × 20.

**Additional file 2: Supplementary Figure 2.** Effect of OSM on the mRNA levels of profibrotic genes in HDMECs. mRNA levels of profibrotic genes were analyzed by quantitative qPCR, *n* = 3. Students t-test *p < 0.05, **p < 0.01 ***p < 0.001.

**Additional file 3: Supplementary Figure 3.** Effect of OSM and IL-6+sIL-6R on migration and proliferation of HDMECs. Migration (**A**) and proliferation (**B**) were examined with the Essen BioScience IncuCyte Live-Cell Imaging system. Data represent *n* = 3 wells for each point with three different cell cultures. *p* <0.05, *p <0.001. **C.** Matrigel tube formation assay of HDMECs stimulated with OSM or IL-6 + sIL-6R.

**Additional file 4: Supplementary Figure 4.** Double IHC staining of PDGFRβ/pSTAT3 and PDGFRβ/PDPN in OSM treated skin cultures. Double IHC staining of PDGFRβ/pSTAT3 (**A**) and PDGFRβ/PDPN (**B**) was performed on paraffin sections from the OSM and IL-6+IL-6Rα treated skin cultures. 25 μm for original magnification × 40 images.

**Additional file 5: Supplemental Table I.** Human primers used for real-time PCR.

**Additional file 6: Supplemental Table II.** Antibodies.

## Data Availability

Datasets related to this article can be found at DOI: 10.17632/5h6w6jjjms.1#folder-762c7894-d8f8-4124-ac6a-f2d22a170c5e

## Abbreviations

αSMA: Alpha smooth muscle actin
CD: Cluster of differentiation
CCL: C-C motif ligand
CHI3L1: Chitinase 3-like protein 1
CXCL: C-X-C motif ligand
dcSSc: Diffuse cutaneous systemic sclerosis
DKK1: Dickkopf WNT signaling inhibitor 1
ECs: Endothelial cells
ECM: Extracellular matrix
EndMT: Endothelial to mesenchymal transition
ERG: ETS-related gene
ET-1: Endothelin 1
FAP: Fibroblast activation protein
FLI1: Friend leukemia integration 1
HAS2: Hyaluronan synthase 2
HDMECs: Human dermal microvascular endothelial cells
ICAM1: Intercellular adhesion molecule 1
IHC: Immunohistochemical
IL: Interleukin
IFN: Interferon
IRB: Institutional Review Board
JUP: Junction plakoglobin
LIFR: Leukemia inhibitory factor receptor
OSM: Oncostatin M
OSMRβ: Oncostatin M receptor beta
PDGFRβ: Platelet-derived growth factor receptor beta
PDPN: Podoplanin
POSTN: Periostin
sIL-6R: Soluble interleukin 6 receptor
SNAIL1: Snail zinc finger 1
SSc: Systemic sclerosis
STAT: Signal transducer and activator of transcription
TGFβ: Transformation growth factor beta
TGFβR: Transformation growth factor beta receptor
TIMP1: TIMP metallopeptidase inhibitor 1
TLR: Toll-like receptor
TNF: Tumor necrosis factor
VCAM-1: Vascular cell adhesion molecule 1
