# Supplementary material for: The role of the oncostatin M/OSM receptor β axis in activating dermal microvascular endothelial cells in systemic sclerosis
Source: Arthritis Res Ther. 2020 Jul 31;22:179. doi: 10.1186/s13075-020-02266-0 (PMC7393919; doi:10.1186/s13075-020-02266-0)
Supplement: Supplementary file 1 — Additional file 1: Supplementary Figure 1. Distribution of OSM in human skin biopsies from healthy controls and SSc patients. A. IHC staining of OSM was performed on paraffin sections from the skin of three SSc patients and three healthy controls 50 μm scale bar for original magnification × 20. [file 13075_2020_2266_MOESM1_ESM.docx]

**Supplementary Figure 1. Distribution of OSM in SSc and healthy control skin biopsies.**
