# Supplementary material for: The role of the oncostatin M/OSM receptor β axis in activating dermal microvascular endothelial cells in systemic sclerosis
Source: Arthritis Res Ther. 2020 Jul 31;22:179. doi: 10.1186/s13075-020-02266-0 (PMC7393919; doi:10.1186/s13075-020-02266-0)
Supplement: Supplementary file 2 — Additional file 2: Supplementary Figure 2. Effect of OSM on the mRNA levels of profibrotic genes in HDMECs. mRNA levels of profibrotic genes were analyzed by quantitative qPCR, n = 3. Students t-test *p < 0.05, **p < 0.01 ***p < 0.001. [file 13075_2020_2266_MOESM2_ESM.docx]

**Supplementary Figure 2. Effect of OSM on the mRNA levels of profibrotic genes in HDMECs.**
