# Supplementary material for: The role of the oncostatin M/OSM receptor β axis in activating dermal microvascular endothelial cells in systemic sclerosis
Source: Arthritis Res Ther. 2020 Jul 31;22:179. doi: 10.1186/s13075-020-02266-0 (PMC7393919; doi:10.1186/s13075-020-02266-0)
Supplement: Supplementary file 3 — Additional file 3: Supplementary Figure 3. Effect of OSM and IL-6+sIL-6R on migration and proliferation of HDMECs. Migration (A) and proliferation (B) were examined with the Essen BioScience IncuCyte Live-Cell Imaging system. Data represent n = 3 wells for each point with three different cell cultures. p <0.05, *p <0.001. C. Matrigel tube formation assay of HDMECs stimulated with OSM or IL-6 + sIL-6R. [file 13075_2020_2266_MOESM3_ESM.docx]

**Supplementary Figure 3. Effect of OSM and IL-6+sIL-6R on migration, proliferation and capillary tube formation of HDMECs**
