# Supplementary material for: The role of the oncostatin M/OSM receptor β axis in activating dermal microvascular endothelial cells in systemic sclerosis
Source: Arthritis Res Ther. 2020 Jul 31;22:179. doi: 10.1186/s13075-020-02266-0 (PMC7393919; doi:10.1186/s13075-020-02266-0)
Supplement: Supplementary file 4 — Additional file 4: Supplementary Figure 4. Double IHC staining of PDGFRβ/pSTAT3 and PDGFRβ/PDPN in OSM treated skin cultures. Double IHC staining of PDGFRβ/pSTAT3 (A) and PDGFRβ/PDPN (B) was performed on paraffin sections from the OSM and IL-6+IL-6Rα treated skin cultures. 25 μm for original magnification × 40 images. [file 13075_2020_2266_MOESM4_ESM.docx]

**Supplementary Figure 4.Double IHC staining of PDGFRb/pSTAT3 and PDGFRb/PDPN in OSM treated skin cultures.**
