# Supplementary material for: The role of the oncostatin M/OSM receptor β axis in activating dermal microvascular endothelial cells in systemic sclerosis
Source: Arthritis Res Ther. 2020 Jul 31;22:179. doi: 10.1186/s13075-020-02266-0 (PMC7393919; doi:10.1186/s13075-020-02266-0)
Supplement: Supplementary file 5 — Additional file 5: Supplemental Table I. Human primers used for real-time PCR. [file 13075_2020_2266_MOESM5_ESM.docx]

**Supplemental Table I. Human primers used for real-time PCR**

|  | Forward 5′ to 3′ | Reverse 5′ to 3′ |
| --- | --- | --- |
| IL-6 | agcctcccagtgcaagattc | caatggcaatgcagaggagc |
| IL-33 | ccaccaaaaggccttcact | aaggcaaagcactccacagt |
| IL-1Ra | tcggctcctagggctctc | cagagggtgcgtctacctg |
| CCL7 | gaaagcctctgcagcacttc | aatctgtagcagcaggtagttgaa |
| CXCL12 | ccaaactgtgcccttcagat | ctttagcttcgggtcaatgc |
| CXCL2 | cccatggttaagaaaatcatcg | cttcaggaacagccaccaat |
| ERG | ccagtcgaaagctgctcaa | gttggtccaagaatctgataagg |
| FLI1 | aaccgggtcaatgtgtggaa | caccgacagagcctccttaat |
| OSMRβ | tgagtttttcatcactccattca | gatatgaatcagcatcgaggagt |
| ICAM-1 | gggagcttcgtgtcctgtat | acttgagctcgggcaatg |
| JUP | gatcttccggctcaacacc | gatgttctccaccgacgagt |
| CAV-1 | acagcccagggaaacctc | cggatgggaacggtgtag |
| SNAIL1 | gctgcaggactctaatccaga | atctccggaggtgggatg |
| TGFβ3 | aagaagcgggctttggac | cgcacacagcagttctcc |
| TGFβR3 | gatttcatcttcggcttgaaa | gctcaggaggaatagtgtgga |
| FGFR1 | accaaaccgtatgcccgtag | cccactggaagggcatttga |
| ET-1 | ttgagatctgaggaacccgc | gagctcagcgcctaagactg |
| FAP | tggcgatgaacaatatcctaga | atccgaacaacgggattctt |
| POSTN | gaaccaaaaattaaagtgattgaagg | tgacttttgttagtgtgggtcct |
| CHI3L1 | cccaacctgaagactctcttgt | ggtgttggaggctatcttgg |
| TIMP1 | gggcttcaccaagacctaca | tgcaggggatggataaaca |
