# Supplementary material for: The role of the oncostatin M/OSM receptor β axis in activating dermal microvascular endothelial cells in systemic sclerosis
Source: Arthritis Res Ther. 2020 Jul 31;22:179. doi: 10.1186/s13075-020-02266-0 (PMC7393919; doi:10.1186/s13075-020-02266-0)
Supplement: Supplementary file 6 — Additional file 6: Supplemental Table II. Antibodies. [file 13075_2020_2266_MOESM6_ESM.docx]

**Supplemental Table II. Antibodies**

| Primary Ab used for WB | Dilution |
| --- | --- |
| Mouse monoclonal Ve-Cadherin (Santa Cruz, Dallas, TX) | 1:1000 |
| Mouse CD31 (PECAM1) (Santa Cruz, Dallas, TX) | 1:1000 |
| Rabbit monoclonal α-Smooth Muscle Actin (Cell signaling, Danvers, MA) | 1:1000 |
| Mouse TGFβ123 (R&D Systems, Minneapolis, MN) | 1:1000 |
| Rabbit STAT3 (Cell Signaling, Danvers, MA) | 1:1000 |
| Rabbit pSTAT3 (Cell Signaling, Danvers, MA) | 1:1000 |
| Mouse β-actin (Sigma, St Louis, MO) | 1:5000 |
| Primary Ab used for IHC | Dilution |
| Mouse anti-human OSMRβ (Santa Cruz, Dallas, TX) | 1:200 |
| Rabbit anti-human OSM (ThermoFisher, Waltham, MA) | 1:200 |
| Rabbit anti-human CD31 (Abcam, Cambridge, UK) | 1:100 |
| Rabbit anti-human pSTAT3 (Cell Signaling, Danvers, MA) | 1:100 |
| Rabbit anti-human PDGFRβ (Cell Signaling, Danvers, MA) | 1:100 |
| Primary Ab used for IF | Dilution |
| Mouse anti-human OSMRβ (Santa Cruz, Dallas, TX) | 1:200 |
| Mouse anti-human VE-cadherin (Santa Cruz, Dallas, TX) | 1:100 |
| Rabbit anti-human OSMRβ (GeneTex, Irvine, CA) | 1:100 |
| Mouse anti-human CD31 (Invitrogen, Carlsbad, CA) | 1:50 |
| Goat anti-human αSMA (Abcam, Cambridge, UK) | 1:100 |
